# Migration routes of three closely related species lead to different non-breeding regions with contrasting environmental conditions

**DOI:** 10.1186/s40462-026-00643-z

**Published:** 2026-03-27

**Authors:** Yann Rime, Dave Lutgen, Áron Péter, Niloofar Alaei Kakhki, Marc Illa, Joanna B. Wong, Gilles Hauser, Marco Pilati, Pavlos Andriopoulos, Sissel Sjöberg, Attila D. Sándor, Reto Burri

**Affiliations:** 1 Swiss Ornithological Institute, Sempach, Switzerland; 2 FitzPatrick Institute of African Ornithology, University of Cape Town, Rondebosch, South Africa; 3 Institute of Ecology and Evolution, University of Bern, Bern, Switzerland; 4 Department of Parasitology and Zoology, University of Veterinary Medicine, Budapest, Hungary; 5 Department of Biodiversity Monitoring, Stuttgart State Museum of Natural History, Stuttgart, Germany; 6 Catalan Ornithological Institute, Barcelona, Spain; 7 Cory’s SCCL, Barcelona, Spain; 8 Department of Biology, Lund University, Lund, Sweden; 9 Department of Biology, National and Kapodistrian University of Athens, Athens, Greece; 10 HUN-REN Climate Change: New Blood-Sucking Parasites and Vector-Borne Pathogens Research Group, Budapest, Hungary; 11 STAR-UBB Institute, Babes-Bolyai University, Cluj-Napoca, Romania

**Keywords:** *Oenanthe hispanica*, *Oenanthe melanoleuca*, *Oenanthe pleschanka*, Long-distance migration, Barrier-crossing, Migratory detour, Range expansion, Non-breeding site ecology

## Abstract

Understanding migration strategies in closely related species is essential to assess population-specific threats and to identify the ecological drivers of speciation. To compare migration strategies and environmental conditions across the annual cycle, we tracked Western Black-eared Wheatears, Eastern Black-eared Wheatears and Pied Wheatears. Individual migration routes were reconstructed from multi-sensor loggers and light-level geolocator data. Western Black-eared Wheatears breeding in Spain migrated south-west to Mauritania and Mali, overwintering at a single site in arid regions at the Sahara’s edge. Eastern Black-eared Wheatears breeding in Greece crossed the Mediterranean and Sahara to reach the Sahel, using single non-breeding sites in Nigeria, Chad, and Niger, much further west than previously assumed. Pied Wheatears breeding in Romania migrated via Turkey and the Arabian Peninsula, and overwintered in Somalia, with some individuals shifting sites during winter. Migration strategies differed: Eastern Black-eared Wheatears performed large-scale loop migration. Pied Wheatears used direct routes and did not follow their presumed former range expansion around the Black Sea. Environmental data suggested that non-breeding conditions contrasted stronger than breeding conditions: Western Black-eared Wheatears occupied arid non-breeding sites at the edge of the Sahara that remained dry throughout their stay, Eastern Black-eared Wheatears arrived in the Sahel at the end of the rainy season, and Pied Wheatears had two rainy seasons in the Horn of Africa. These results highlight distinct migration strategies and non-breeding conditions in three closely related wheatears, underlining the importance of species-specific or even population-specific tracking to refine non-breeding range delineation and inform conservation.

## Introduction

Unravelling the spatial and temporal processes involved in shaping bird migration is crucial to understand the full annual cycle of migrant species [35]. In particular, the global decline of long-distance migrants calls for research investigating their migration behaviour [10, 33, 44, 45, 52]. While the species-wide breeding and non-breeding ranges of Palaearctic birds are often broadly described based on observational data, exact migration routes often remain difficult to disentangle, especially for species with low detection rates or challenging identification. Yet, especially for small, isolated or declining populations of a species, information on population-specific migration patterns is often highly relevant to understand year-round threats to population persistence. Furthermore, the links between population-specific migration routes and their evolutionary history and range expansion remain poorly understood [49, 59]. To start filling these research gaps, individual tracking enables the identification of population- and species-specific migration routes [16, 42, 43].

The evolution of the most extreme and fascinating migration strategies derives from range expansions. These are typically present among the *Oenanthe* genus. Migration routes of Northern Wheatears *Oenanthe oenanthe*, for example, display a stunning diversity that is thought to reflect the colonization history of the species’ circumpolar distribution: non-breeding sites were conserved after the species’ range expanded. This led to populations from Canada and Greenland that overwinter in West Africa, while populations from Alaska migrate to East Africa [7, 12, 46]. Whether this phenomenon is found in other species of the *Oenanthe* genus remains unknown. In other taxa, migration routes often follow range shift or expansion, with the example of Arctic Warblers *Phylloscopus borealis* migrating eastwards through Siberia before heading south towards south-east Asia [20]. This route follows the ancestral range expansion and avoids the Himalayas as a major migratory barrier, a behaviour that is also observed in other Siberian species [58]. While migration patterns are often conserved within bird species, they can also change within a relatively short time frame, leading to fast evolution of new migration routes [55].

Although studies on passerines have provided valuable insights into variation in migration routes among populations of the same species and in their degree of migratory connectivity (e.g. [22, 24, 31, 46]), comparisons of migration strategies in closely related species remain rare. Here, we investigate migration routes of three wheatear species. Western Black-eared Wheatears *Oenanthe hispanica*, Eastern Black-eared Wheatears *Oenanthe melanoleuca* and Pied Wheatears *Oenanthe pleschanka* are closely related species that diverged less than 700’000 years ago [30]. Western Black-eared Wheatears breed in the western Mediterranean, while Eastern Black-eared Wheatears breed in the Middle East and the Eastern Mediterranean, including the Balkan peninsula, reaching northern Bulgaria where the range narrowly overlaps with a small, isolated population of the Pied Wheatear, whose distribution in this region is restricted to the Pontic steppes along the Black Sea shores [13, 14]. The Pied wheatears found in this region (“Pontic lineage”) have recently been shown to be genetically distinct from ones in the species’ Asian range [30]. The other populations of Pied Wheatear expanded throughout central Asia to China, all overwintering in the Afrotropic. Recent genomic studies highlighted that the Pontic region was colonized by Pied Wheatears from further east, likely along the northern shore of the Black Sea [29]. Eastern Black-eared and Pied Wheatears hybridize in several contact zones, including the Pontic region [30, 40, 47]. Observational data indicates that Western Black-eared Wheatears seem to overwinter only in the western Sahel, while Eastern Black-eared Wheatears overwinter in the eastern Sahel, supposedly east of Lake Chad [14, 60]. As females of the two Black-eared Wheatear species are very similar and almost cryptic and also males feature very similar non-breeding plumage, the delineation and potential overlap of non-breeding ranges are still unclear. The non-breeding range of Pied Wheatears is mostly in the Horn of Africa [13], but the migration routes of the different populations are not known, yet. Furthermore, observational data coverage in the non-breeding range remains insufficient to assess the environmental conditions encountered by the three species in winter, and whether these differ between species.

With this study, we aimed to provide an overview of the migration routes of three closely related Wheatear species breeding in Southern Europe. We investigated barrier-crossing strategies, i.e. whether they would cross the Mediterranean Sea or circumvent it. Furthermore, we evaluated if the environmental conditions encountered during the annual cycle differed between species, both at breeding and non-breeding sites, providing insight in potential ecological drivers of speciation. For Eastern Black-eared Wheatears, we asked if the non-breeding range of the Balkan population corresponds to the description in the literature [14] or if it overlaps with the range of Pied and Western Black-eared Wheatear. For the Pontic population of the Pied Wheatear, we aimed to determine whether the migration route follows the putative post-glacial colonisation, which for individuals breeding in Romania would likely involve flying east around the northern shore of the Black Sea (and potentially the Caspian), before heading south, or if they have adjusted to the most direct route.

## Materials and methods

### Study areas and study design

Western Black-eared Wheatears were captured and equipped with multi-sensor loggers in northwestern Spain at 41.83 N, 1.32 E and 41.84 N, 1.33 W. Nine individuals were equipped with multi-sensor loggers in July-August 2024 whereof five individuals were resighted, and three individuals (males) were recaptured in the following year. Eastern Black-eared Wheatears were captured in Greece at Distomo (16 individuals, 38.43 N, 22.64 E), Itea (seven ind., 38.42 N, 22.61 E), Galaxidi (five ind., 38.41 N, 22.33 E), Livadi Arachovas (1 ind., 38.49 N, 22.55 E) and Pantes (six ind., 38.38 N, 22.27 E) in May-June 2022. 24 individuals were equipped with light-level geolocators, and 11 individuals were colour-ringed as a control group. We resighted eight individuals with geolocators in 2023, four of which (males) could be recaptured successfully, as well as two control individuals. Pied Wheatears were captured at the Sitorman quarry in Eastern Romania’s Dobrogea Region (31 ind., 44.42 N, 28.52 E) and Cheia (2 ind., 44.51 N, 28.43 E). 22 individuals were equipped with light-level geolocators in May-June 2022 and 11 birds served as a control group. We resighted five tagged individuals in 2023, from which four (males) were recaptured and provided data. Three colour-ringed control birds were resighted. The study areas in Spain and Greece are characterized by a warm, Mediterranean climate with open landscape and low, shrubby and sparse Mediterranean vegetation. The Romanian sites have a continental climate with hot summers, and shortgrass steppe vegetation. The habitat in the Sitorman quarry is significantly rockier compared to the surrounding agricultural land and hosts the most important Pied Wheatear breeding population in the country.

Birds were captured and recaptured using mist-nets, spring traps baited with meal worms or perch traps where they were lured with playback. Every bird was ringed with an aluminium ring from the country’s ringing scheme and with a unique colour-ring combination. Multi-sensor loggers custom-made from Lund University were used to track the migratory route of Western Black-eared Wheatears. These loggers included an accelerometer (to approximate activity; 10 min resolution), a temperature compensated pressure sensor (1 h resolution) and a light-level sensor (5 min resolution) and weighted ~ 0.8 g (for detailed description of the function and measurement scheme of the multi-sensor loggers see [6, 48]). One individual’s logger failed at the end of post-breeding migration in Mauritania. To track the migratory routes of four Eastern Black-eared and four Pied Wheatears from Greece and Romania, we used GDL-2 light-level geolocators from the Swiss Ornithological Institute (0.6 g, recording light intensity every 5 min). All devices were mounted on a leg-loop harness [41] and birds were released within 20 min after capture. Loggers weighed between 3 and 4% of the birds’ body mass. Return rates of Western Black-eared Wheater equipped with 0.8 g loggers were higher than in previous studies on Wheatears [5, 31, 43]. Compared to the return rates of Western Black-eared Wheatears, return rates were lower in Pied and Eastern Black-eared Wheatears (equipped with 0.6 g loggers), but these return rates were not lower than those of control birds. Given the small sample size and lack of correlation with logger weight, we cannot attribute a difference in return rate to the loggers. Difficulty to resight the birds, or differences in site fidelity or general survival might be at play.

### Multi-sensor logger data analyses

To analyse data from multi-sensor loggers for Western Black-eared Wheatears (*n* = 3), we modelled trajectories from pressure and wind data using the R Package GeoPressure R [36] following the procedure described in Nussbaumer et al. [38, 39]. We summarize the main steps here: 1) we identified migratory flights by combining acceleration data (high activity values) and pressure data (sudden change in pressure indicating altitudinal movement). Periods between migratory flights were defined as stationary locations. 2). We then built pressure likelihood maps by matching geolocator barometric measurements during each stationary period with data from the ERA5 reanalysis [21]. Finally, we modelled individual trajectories with a hidden Markov model based on pressure likelihood maps as well as wind data. From this model, we produced a probability map for the position at each stationary period, the most likely individual path for each bird and 100 random simulations of the trajectories. Multi-sensor logger data for Western Black-eared Wheatear are available at https://doi.org/10.5281/zenodo.18755138.

### Light-level geolocator data analyses

To analyse light-level geolocator data for Eastern Black-eared (*n* = 4) and Pied Wheatears (*n* = 4), we first identified twilight events (sunrises and sunsets) using the package TwGeos [28]. The different stationary periods were then identified using a threshold method where stark changes in successive twilights (that is, at midday and midnight) indicate a change of location. Stationary periods were defined as stops lasting at least two days [28]. We selected a light intensity threshold of one unit on the log-transformed scale. We then used the R Package GeoLight [27] to perform an “in-habitat” calibration at the breeding site. Further analyses were conducted following a Bayesian framework using the R package SGAT, to obtain the most likely median migration paths [56]. We used the Group Estelle model (“groupedThresholdModel” function) to estimate a set of coordinates with a 95% credible interval for each stationary period based on the twilights recorded at this location. We assumed the error distribution of twilight events to follow a Gamma distribution based on the parameters from the calibration. We also included a land mask to give locations on land a higher prior. Around equinox periods, when day and night lengths are equal, the solar declination angle is close to 0°, resulting in inaccurate latitude estimates. To account for this, we interpolated over the equinox periods using a tolerance on the sine of the solar declination angle between 0.04 and 0.097 (corresponding to ~ 5.7 to 13.9 days before and after the equinoxes). To identify the most likely stationary locations, we applied a speed distribution (Gamma distributed: shape = 2.2, scale = 0.06). The highest probability of ground speed was defined between 10 and 40 km h^− 1^ during the movement phase. We attributed a fixed location to the equipment and retrieval sites. To initiate the model, we first used a “modifiedGamma” model with relaxed assumptions for 1000 iterations. We then tuned the model five times, with 300 iterations each, with all priors from the “Gamma” model, before a final run of 2000 iterations to improve convergence. We show the most likely path and stationary sites as the median coordinate estimates and their 95% probability distributions [38].

### Range maps

We downloaded range maps of all three species from BirdLife International and Handbook of the Birds of the World [9]. For Western and Eastern Black-eared Wheatears, the maps reflects the latest taxonomic revision in the range maps in Birds of the World online [14, 15].

### Environmental data

To investigate environmental conditions encountered by individuals at the breeding and non-breeding sites and ultimately compare the conditions used by the three species, we first defined the main non-breeding site as the longest stationary period during the boreal winter and extracted environmental data from this time frame using the Google Earth Engine [19]. To account for spatial imprecision of the light-level geolocation, all data were extracted in a 100 km radius at the breeding site and around the most likely location for the non-breeding site. We refrained from conducting similar analyses for stopover sites, where the uncertainty is higher. Precipitation and subsequent vegetation greening influence the availability of food resources and the habitat characteristics encountered by birds [32, 60]. To inform on these environmental conditions at the non-breeding sites, we extracted the precipitation data from the CHIRPS daily dataset [18], the daily mean temperature data from ERA-5 reanalysis data [34], and we used the Normalized Difference Vegetation Index (NDVI) data from Harmonized Sentinel-2 MultiSpectral Instrument with a five-day resolution [17]. On coastal regions, we excluded areas that were not on land from the areas for which environmental variables were considered.

## Results

**Fig. 1:**
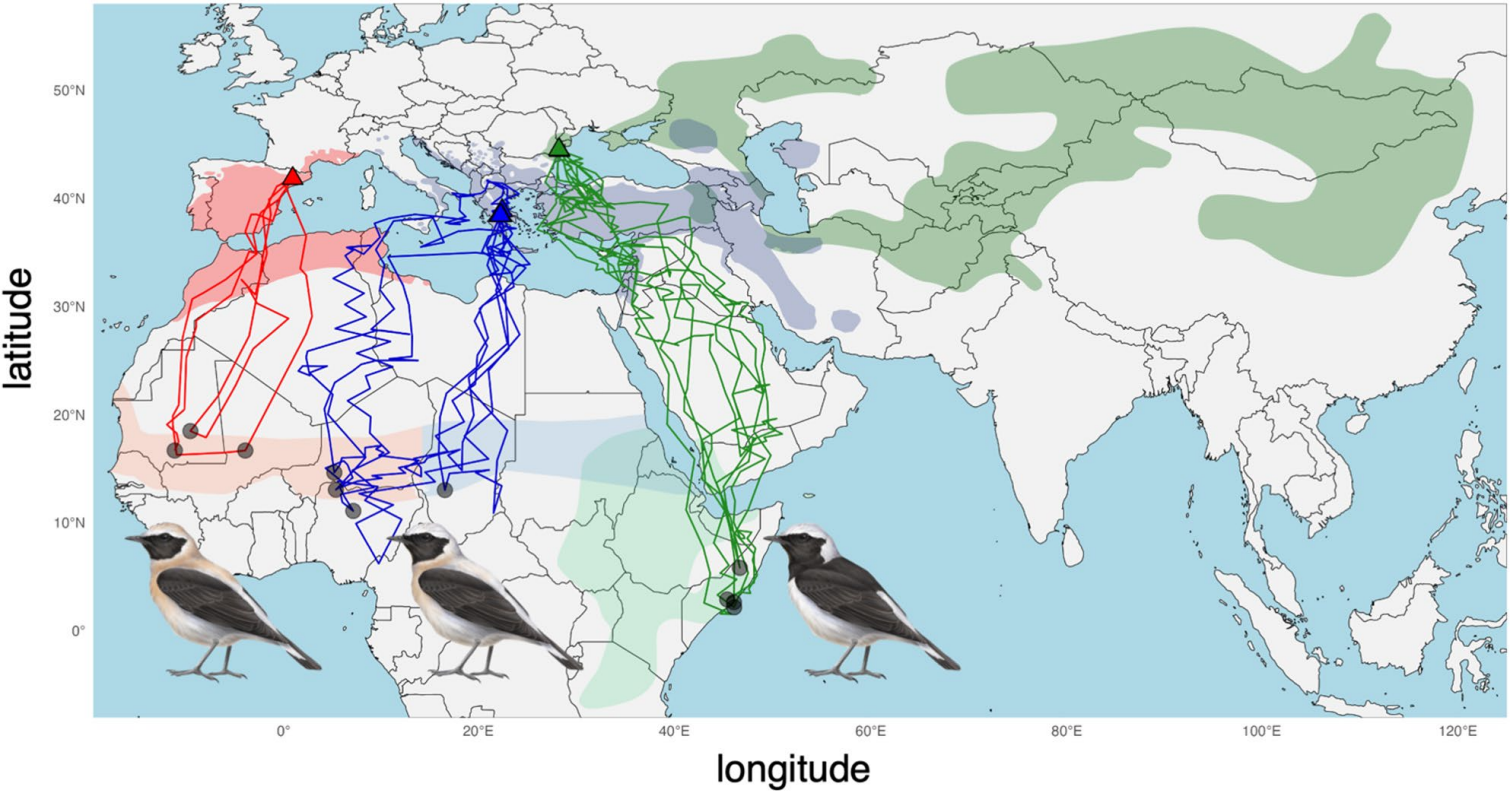
Migration routes of Western Black-eared Wheatears from Spain (red lines), Eastern Black-eared Wheatears from Greece (blue lines) and Pied Wheatears from Romania (green lines). The main individual non-breeding sites in Africa are highlighted as a black dot. Following the same colours as the migratory tracks, dark shaded areas show the breeding distribution of the three species and the light shaded areas show the non-breeding distribution according to BirdLife International and Handbook of the Birds of the World [9]. This highlights that Eastern Black-eared Wheatears overwinter much further west than expected in the western Sahel, within the expected range of Western Black-eared Wheatears, while the westernmost population of Pied Wheatears reaches the easternmost side of the non-breeding distribution in Somalia. Illustrations © Manuel Schweizer

**Fig. 2:**
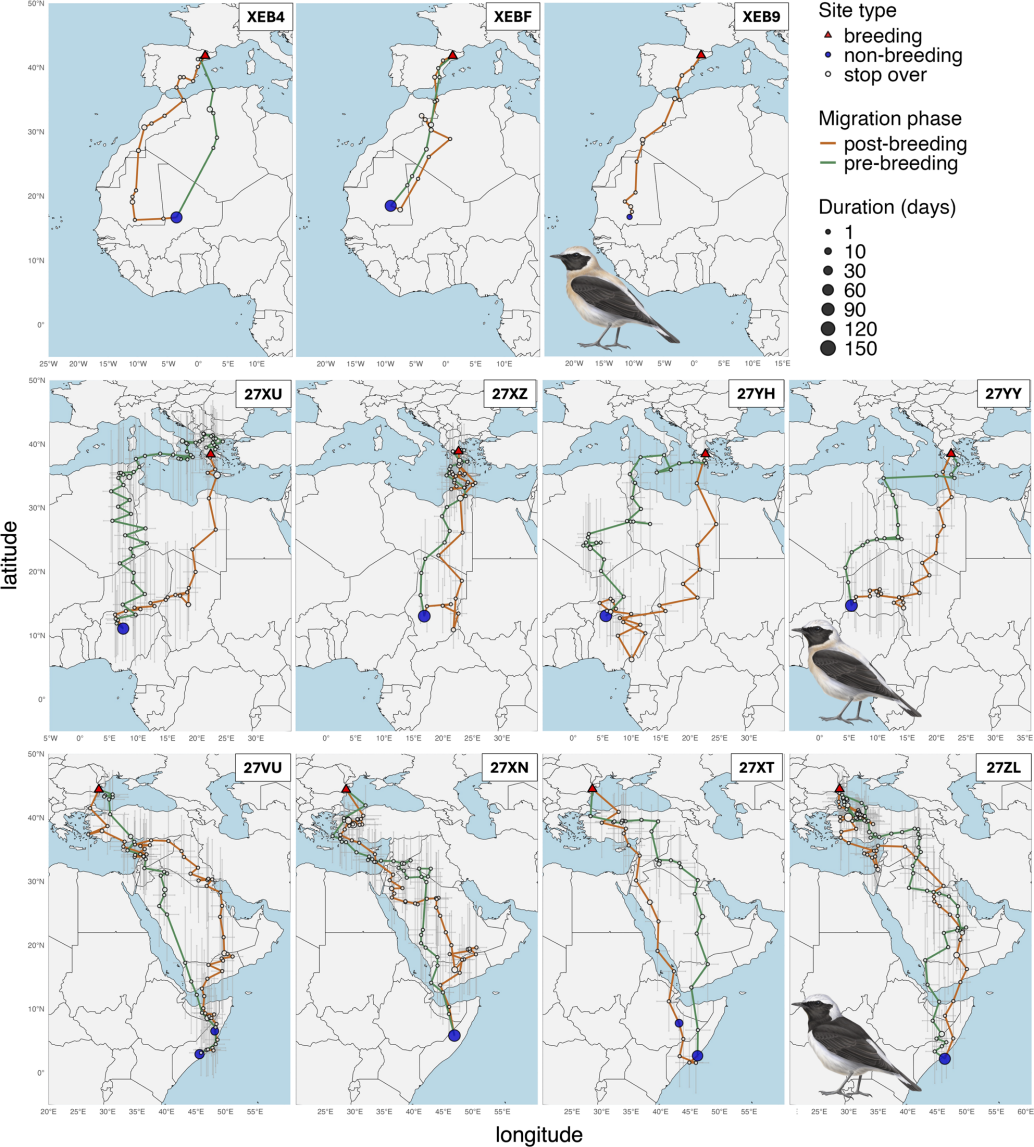
Migration routes of the three studied species (from top row: Western Black-eared, Eastern Black-eared, and Pied Wheatear). Red triangles show the breeding sites, blue dots show the non-breeding sites, and empty dots show stopover sites, with dot size proportional to the duration of stay. The orange line shows the post-breeding migration, while the pre-breeding migration appears in green. For Western Black-eared Wheatears, barometric data enabled higher geographic precision, and we only plotted the most likely route. For light-level geolocation (Eastern Black-eared and Pied Wheatears), the uncertainty (standard deviation of each stationary location) is represented as grey vertical bars (latitudinal uncertainty) and horizontal bars (longitudinal uncertainty) for light-level geolocation data. Illustrations © Manuel Schweizer

**Fig. 3:**
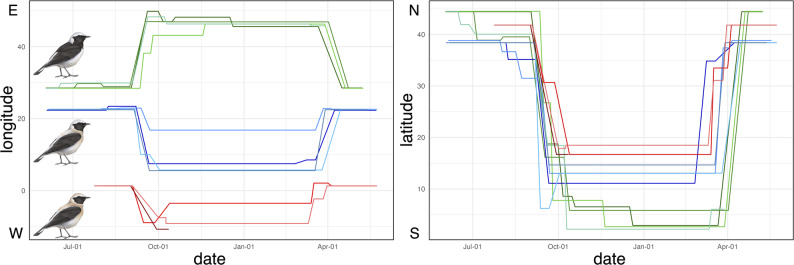
Longitudinal (left) and latitudinal (right) patterns of migration of Western Black-eared (red), Eastern Black-eared (blue) and Pied Wheatears (green) accounting for stopovers of at least 7 days. Illustrations © Manuel Schweizer

Western Black-eared Wheatears from Spain migrated south-westwards to Mauritania. One individual remained in Mauritania for the entire winter, and one individual reached a single non-breeding site in Tombouctou region of Mali (Figs. 1, 2 and 3). All individuals crossed the Mediterranean Sea in the west, during both post- and pre-breeding migrations, but did not take a detour through the strait of Gibraltar to minimize migration over water (Fig. 2).

Eastern Black-eared Wheatears from Greece migrated southwards across the Mediterranean Sea (Figs. 1 and 2) and then crossed the Sahara to reach the Sahel region at the end of the rainy season. After several stopovers south of the Sahara, each individual used a single non-breeding site. The main non-breeding sites were located in northern Nigeria, southern Chad, and south-eastern Niger, much further west than the previously described non-breeding range (Fig. 1). Compared to post-breeding migration, pre-breeding migration occurred further west (Fig. 2). It included direct sea crossing, likely from Tunisia to Greece in three individuals, suggesting that individuals did not minimize flights over water. A fourth individual took a spring route similar to the route used during post-breeding migration (Fig. 2).

Pied Wheatears from Romania all took the shortest route towards Turkey and did not undertake a detour along the Black Sea (Figs. 1 and 2). In contrast to Eastern Black-eared Wheatears, they avoided the Mediterranean altogether by flying eastwards over Turkey or flew only over a much shorter stretch of Sea compared to Eastern Black-eared Wheatears. They then migrated across the Arabian Peninsula before reaching the Horn of Africa in central Somalia, where some birds changed location during winter (Figs. 1 and 2). Pre-breeding migration was more direct, with no stopover longer than seven days (Figs. 2 and 3).

**Fig. 4:**
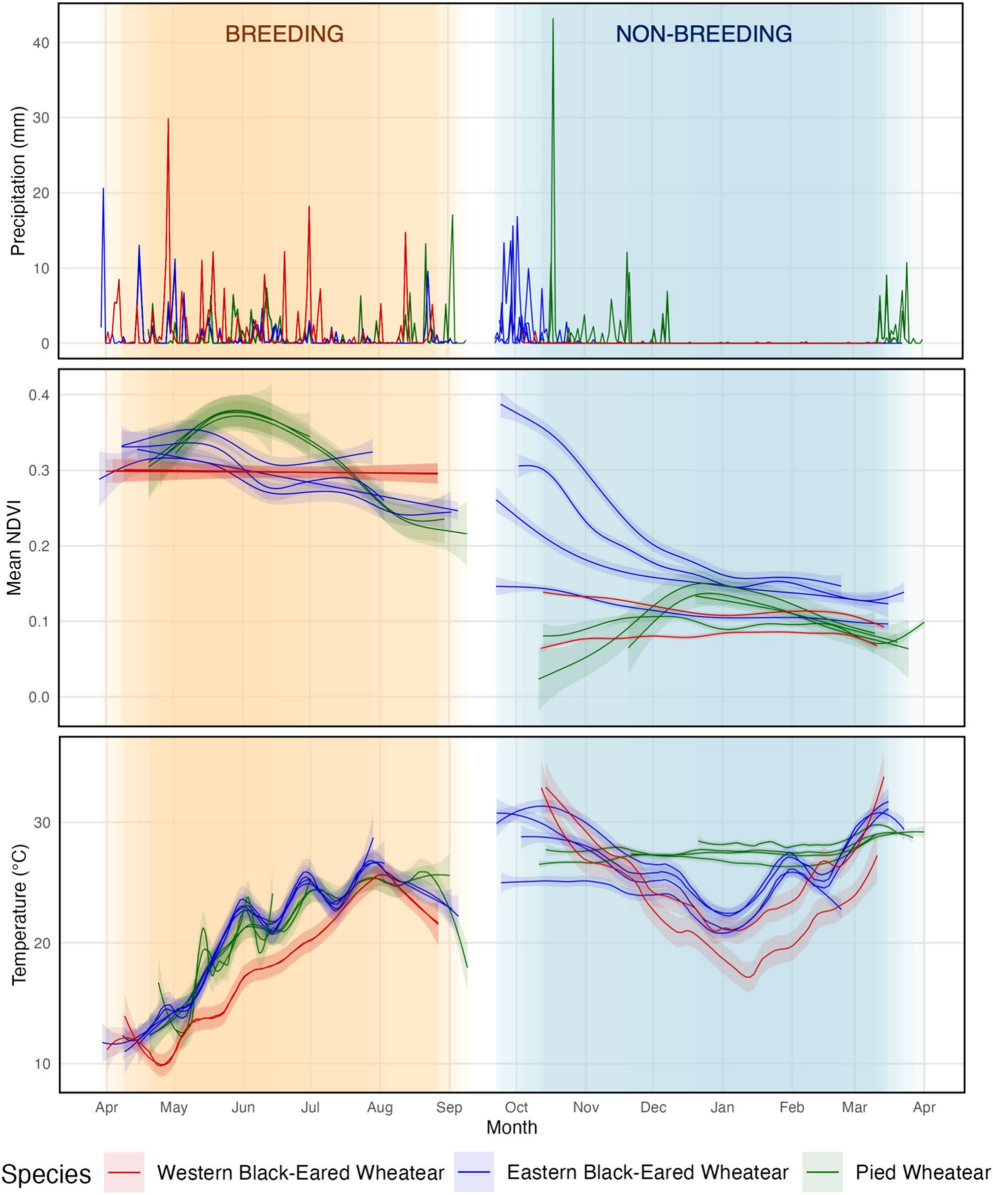
Pattern of precipitation (raw data on a 100-km radius), NDVI (normalized difference vegetation index, aggregated mean on a 100-km radius fitted in a general additive model), and temperature at breeding sites and at the longest non-breeding site for Western Black-eared Wheatear (red line), Eastern Black-Eared Wheatear (blue line) and Pied Wheatear (green line)

The analysis of remote-sensing data (Fig. 4) suggests that environmental conditions encountered at breeding sites were similar among the three species in terms of temperature and rainfall. However, breeding sites differed in terms of vegetation greening, with stable NDVI in Spain between April and July and earlier spring onset in Greece compared to Romania, where vegetation greening peaked later in the breeding season. Differences were even more marked in the non-breeding regions. Western Black-eared Wheatears overwintered in very arid areas at the edge of the Sahara, without rainfall and with low NDVI even at the start of the non-breeding season. Eastern Black-eared Wheatears arrived on their single non-breeding grounds in the Sahel region at the end of the rainy season when vegetation greening was decreasing. Temperatures were generally moderate and declined during most of the stationary non-breeding periods of both Western and Eastern Black-eared Wheatears. In contrast, Pied Wheatears encountered two rainy periods, with vegetation greening peaking during their stay and more stable and constantly relatively high temperatures (Fig. 4).

## Discussion

Understanding migrating birds’ non-breeding ranges is often difficult based on observational data alone. This challenge is pronounced for cryptic species that are difficult to identify and migrate to regions that are remote or difficult to access for security reasons. This is further reflected in the different non-breeding areas uncovered here, which challenges the previously assumed wintering locations based on observational data. Our geolocation data show that Eastern Black-eared Wheatears overwinter more than 1000 km further west than reported in the literature, which limited the non-breeding range of the species to east of Lake Tchad [14, 60]. The uncovered non-breeding sites involves potential overlap with the non-breeding range of Western Black-eared Wheatears, calling for more careful identification of these species in the field [15]. In Pied Wheatears, we show that all four tracked individuals spent the non-breeding season in the south-easternmost part of the known non-breeding range in Somalia, even though the species also occurs in the Eastern Sahel, closer to the breeding sites [13]. What remains unclear is whether this also reflects in differing non-breeding ranges between Asian populations of Pied Wheatears and the recently genetically uncovered Pontic lineage of this species [30] tracked here, and how environmental conditions may differ between these ranges.

Migration patterns also showed important variation between the studied species in terms of movement strategies. Loop migration, as performed by the tracked Eastern Black-eared Wheatears, is a phenomenon that has been documented in other Afro-Palearctic migrants where birds do not follow the same longitudinal path in spring and in autumn. Typical drivers of loop migration patterns are wind support and the availability of favourable conditions along the way, or a mix of both [1, 11, 24]. Meanwhile, loop migration patterns were either absent or unclear in the tracks of Western Black-eared or Pied Wheatears, and their migration routes did not seem to follow similar patterns as those of Cyprus Wheatears, which reach Sudan directly from the breeding site in Cyprus in the post-breeding migration and return further east on their longer pre-breeding migration [57]. Moreover, the three species also responded differently to open water crossings. Western Black-eared Wheatears crossed a short extent of the western Mediterranean, despite a possible shorter sea crossing through the strait of Gibraltar, while Eastern Black-eared Wheatears crossed the Mediterranean over large stretches, similar to Cyprus Wheatears [57] and Northern Wheatears [31, 43]. Pied Wheatears mostly flew over land east of the Mediterranean, although short flights over the sea likely occurred. Given the uncertainty of locations provided by light-level geolocators and because of the two-days stopover threshold, caution is required in interpreting barrier crossing in the Eastern Black-eared and Pied Wheatear data. It is unclear whether Eastern Black-Eared Wheatears used islands as stopover sites during their Sea crossing, as Northern Wheatears sometimes do [43]. While the general migratory direction in the three closely related species is likely influenced by geography, our results suggest interspecific variation of migration patterns that cannot be explained by geography alone. This complexity calls for more comprehensive investigation of migration behaviours in closely related species and within different populations of the same species.

Migration routes often reflect a species’ evolutionary history, where species such as Arctic Warblers or Northern Wheatears do not follow direct routes from breeding to non-breeding grounds and do not reach the closest available non-breeding habitats, but rather follow historical colonisation routes [3, 7, 20]. In contrast, here, Pied Wheatears did not follow the hypothesized routes of colonization along the northern shores of the Black Sea but migrated directly through Anatolia and the Arabian Peninsula. Information on migration routes of Asian populations of Pied Wheatears are still lacking. Larger sample sizes and further data on other populations of these species are still needed to infer the degree of migratory connectivity across populations [4, 54]. Further investigation should also include representative samples of the different age classes and sexes, which was not possible here given the difficulty to capture females and the low return rates of juveniles. Including additional sensors in future studies, such as barometric sensors used here for Western Black-eared Wheatears [37, 39, 42], will inform more precisely on migration behaviour and enable the inference of stationary sites with more precision.

Despite our limited sample sizes and the limits of tracking only one population per species in different years, consistent results between individuals suggest distinct environmental conditions encountered by the three species throughout their annual cycles. Differences were present at breeding sites, with more stable vegetation conditions in Spain and typically earlier spring onset in Greece compared to Romania, reflected by a delayed peak in vegetation greening for Pied Wheatears. Even though Eastern Black-eared Wheatear arrived only a few days before Pied Wheatears to their breeding sites, the contrasting spring conditions encountered by both species might explain the current range of the species and drive future changes in distribution. Temperature was similar and increased at the breeding site of all three species over the breeding season and no clear difference in precipitation patterns were noted. Overall, differences in environmental conditions between species were more contrasting at the non-breeding sites. Conditions at non-breeding sites also differed from those at breeding sites, highlighting a major difference between breeding and non-breeding niche in all three species. Western Black-eared Wheatears overwintered at higher latitudes in very arid areas at the limit between the Sahel region and the Sahara Desert. Therefore, they did not benefit from fluctuations in vegetation greening and related peaks in food abundance. Eastern Black-eared Wheatears reached more humid areas in the Sahel relatively directly in autumn at the end of the rainy season, when vegetation greening and food availability are still high. Many species, including birds connected with wetlands, move over large distances during the non-breeding season to track local conditions [24–26, 50, 51]. However, the tracked individuals of Western and Eastern Black-eared Wheatears stayed at the same site for the entire non-breeding season, a behaviour similar to Northern Wheatears [43] which use the same habitat patch over the entire dry season. In contrast, two of four Pied Wheatears used several sites but always within the same region in the Horn of Africa, where they benefitted from two periods of rain and more stable temperatures throughout their stay. More data would be needed to better understand the causes of these movements within the non-breeding range. Furthermore, the potential overlap of non-breeding ranges between both Black-eared Wheatear species calls for further investigation regarding niche differentiation within the shared range.

This study combines tracking methods to provide information on the migration strategies of three closely related bird species, challenging previous observation-based assumptions. Our results highlight presence of Eastern-Black-eared Wheatears further West than expected, in the range of their Western counterparts. Moreover, they shed light on the contrasting ecological conditions encountered at wintering sites. Such conditions are likely to be impacted by current climatic and land-use changes in the Sahel and the Horn of Africa [2, 8]. While Western Black-eared Wheatears are locally declining in their breeding range [23], it is still unclear what impact changes in the non-breeding grounds have on the populations of these species, confirming the widely acknowledged need for more research on birds overwintering south of the Sahara [53]. Finally, our results suggest that alongside habitat and environmental conditions at the breeding sites, non-breeding conditions are also likely to have played a crucial role in shaping the current range of the species.

## Data Availability

Data and code used in this work are available at 10.5281/zenodo.18757344. Western Black-eared Wheatear multi-sensor data are also archived as a data package at 10.5281/zenodo.18755138.
